# Supplementary material for: Phase-separated NDF−FACT condensates facilitate transcription elongation on chromatin
Source: Nat Cell Biol. 2025 Sep 30;27(11):1938–51. doi: 10.1038/s41556-025-01778-8 (PMC12611769; doi:10.1038/s41556-025-01778-8)
Supplement: Supplementary file 1 — Supplementary Tables 1 and 2: nucleosomal DNA sequences and summary of NDF-WT and NDF_K161A mutant activities. [file 41556_2025_1778_MOESM1_ESM.pdf]

nature cell biology

Supplementary information https://doi.org/10.1038/s41556-025-01778-8

Phase-separated NDF–FACT condensates facilitate transcription elongation on chromatin

In the format provided by the authors and unedited

Supplementary Table 1. Nucleosomal DNA sequences used in this study

PC 601	ACGAAGCGTAGCATCACTGTCTTGTGTTTGGTGTGTCTGGGTGGTGGC CGTTTTCGTTGTTTTTTTCTGTCTCGAACCTGGAGACTAGGGAGTAATC CCCTTGGCGGTTAAAACGCGGGGGACAGCGCGTACGTGCGTTTAAGC GGTGCTAGAGCTGTCTACGACCAATTGAGCGGCCTCGGCACCGGGAT TCTGAT
Widom601	ACGAAGCGTAGCATCACTGTCTTGTGTTTGGTGTGTCTGGGTGGTGGC CGTACAGGATGTATATATCTGACACGTGCCTGGAGACTAGGGAGTAAT CCCCTTGGCGGTTAAAACGCGGGGGACAGCGCGTACGTGCGTTTAAG CGGTGCTAGAGCTGTCTACGACCAATTGAGCGGCCTCGGCACCGGGA TTCTGAT

**Protein**	**Interaction with FACT**	**Condensate Formation with FACT**	**Catalytic Activity (In vitro)**	**Synergistic Effect with FACT on Transcription**
**Wild-type NDF**	Strong interaction with FACT<br><br>(Figure 1c)	Forms robust condensates in vitro (Figure 2b)<br><br>and<br><br>in cells (Figure 5a)	Effective in aiding Pol II transcription<br><br>(Extended Data Figure 4h-i)	Significant synergy with FACT in vitro (Figure 1e-f)
**NDF_K161, _R162 mutants (Full-length)**	Strong interaction with FACT<br><br>(Figure 4d)	Fails to form stable condensates in vitro (Figures 4e-f)<br><br>and<br><br>significantly reduced puncta in cells (Figure 7i-j)	Effective in aiding Pol II transcription<br><br>(Extended Data Figure 4h-i)	Loses synergy with FACT in vitro (Figures 4g-h)<br><br>and<br><br>shows impaired cell growth, transcription and chromatin defects in cells (Figure 7)

Supplementary Table 2: Summary of NDF_WT and _K161A mutant activity tested in this study.
