# Supplementary material for: Phase-separated NDF−FACT condensates facilitate transcription elongation on chromatin
Source: Nat Cell Biol. 2025 Sep 30;27(11):1938–51. doi: 10.1038/s41556-025-01778-8 (PMC12611769; doi:10.1038/s41556-025-01778-8)
Supplement: Supplementary file 2 — Reporting Summary [file 41556_2025_1778_MOESM2_ESM.pdf]

nature portfolio

Corresponding author(s): Jia Fei, Carlos Bustamante

Last updated by author(s): Aug 8, 2025

# Reporting Summary

Nature Portfolio wishes to improve the reproducibility of the work that we publish. This form provides structure for consistency and transparency in reporting. For further information on Nature Portfolio policies, see our Editorial Policies and the Editorial Policy Checklist.

## Statistics

For all statistical analyses, confirm that the following items are present in the figure legend, table legend, main text, or Methods section.

| n/a | Confirmed | |
|---|---|---|
| ☐ | ☒ | The exact sample size (*n*) for each experimental group/condition, given as a discrete number and unit of measurement |
| ☐ | ☒ | A statement on whether measurements were taken from distinct samples or whether the same sample was measured repeatedly |
| ☐ | ☒ | The statistical test(s) used AND whether they are one- or two-sided<br>*Only common tests should be described solely by name; describe more complex techniques in the Methods section.* |
| ☒ | ☐ | A description of all covariates tested |
| ☒ | ☐ | A description of any assumptions or corrections, such as tests of normality and adjustment for multiple comparisons |
| ☐ | ☒ | A full description of the statistical parameters including central tendency (e.g. means) or other basic estimates (e.g. regression coefficient) AND variation (e.g. standard deviation) or associated estimates of uncertainty (e.g. confidence intervals) |
| ☐ | ☒ | For null hypothesis testing, the test statistic (e.g. $F$, $t$, $r$) with confidence intervals, effect sizes, degrees of freedom and $P$ value noted<br>*Give P values as exact values whenever suitable.* |
| ☒ | ☐ | For Bayesian analysis, information on the choice of priors and Markov chain Monte Carlo settings |
| ☒ | ☐ | For hierarchical and complex designs, identification of the appropriate level for tests and full reporting of outcomes |
| ☒ | ☐ | Estimates of effect sizes (e.g. Cohen's *d*, Pearson's *r*), indicating how they were calculated |

*Our web collection on statistics for biologists contains articles on many of the points above.*

## Software and code

Policy information about availability of computer code

| | |
|---|---|
| Data collection | Paired-end sequencing was conducted using Illumina NovaSeq X Plus.<br>ZEN (version 2.6) imaging software was used for collecting confocal images.<br>Leica Application Suite X (LAS X) was used for collecting FRAP images. |
| Data analysis | For ChIP-seq, Cut&Run, Pro-seq and MNase-seq, the sequencing data were trimmed with Fastp (version 0.23.4) and Trim Galore! (version 0.6.7) Programs and aligned to the hg19 reference genome using Bowtie2 (version 2.5.3). Spike-in reads were mapped to the dm3 genome build. PCR duplicates were removed with the samtools (version 1.15.1). For ChIP-seq, Cut&Run, Pro-seq, the normalized bigwig files and metagene analysis were prepared by Homer (version 4.10) or Deeptools (version 3.5.4). For MNase-seq, the bam files generated by Bowtie2 were further analysis by Danpos (version 2.2.2), the wig files generated by Danpos were converted to bigwig files with UCSC Tool wigToBigWig (v482). The nucleosome coverage an metagene profiles were further generated by Deeptools and normalized base on the reads of 2,437 transcriptionally inactive genes in cluster 4 (Figure 4c). For RNA-seq, the raw sequencing data were trimmed and aligned to the hg19 reference genome and the ERCC Spike-in RNA annotation using Hisat2 (version 2.2.1). Total reads from each gene were calculated using featureCounts (version 2.0.3). Differential gene expression analysis was performed using DESeq2 (v2.11.40.8), and the results were visualized using the EnhancedVolcano (v1.20.0, https://github.com/kevinblighe/EnhancedVolcano) package. For TT-seq, raw sequencing reads were trimmed and aligned to both the human reference genome (hg38) and S. pombe genome using STAR (version 2.7.11b). PCR duplicates were removed using samtools. Mapped BAM files were used to analyze transcriptional wave peaks and RNA polymerase II elongation speed, following previously described protocols with minor modifications. Analyses were restricted to genes longer than 50kb with non-overlapping transcription units (n = 2,558 transcripts in hg38). Gene regions were extended from −2kb upstream to +80kb downstream of the transcription start site (TSS); any extensions beyond chromosome boundaries were excluded.The images were processed and analysis with Fiji (version 2.14.0). For single-molecule data, the raw data was exported from the optical tweezers instruments and converted to .mat files to be analyzed with custom-written Matlab code (https://github.com/abmtong/BLabOTMatlab/archive/refs/heads/master.zip). This code was used |

to align the transcription traces, plot the transcribed distance in bp, define the position of Pol II along the template, generate the transcriptional maps of the nucleosome, and correlate the optical tweezers channel with the single molecule fluorescence channel.

For manuscripts utilizing custom algorithms or software that are central to the research but not yet described in published literature, software must be made available to editors and reviewers. We strongly encourage code deposition in a community repository (e.g. GitHub). See the Nature Portfolio guidelines for submitting code & software for further information.

## Data

Policy information about availability of data

All manuscripts must include a data availability statement. This statement should provide the following information, where applicable:

- Accession codes, unique identifiers, or web links for publicly available datasets
- A description of any restrictions on data availability
- For clinical datasets or third party data, please ensure that the statement adheres to our policy

ChIP-Seq, CUT&RUN, Pro-seq and RNA-Seq data have been deposited at the NCBI gene expression omnibus (GEO; www.ncbi.nlm.nih.gov/geo/) with the GEO accession number GSE273678, GSE273679, GSE273680 and GSE273681 respectively.

## Research involving human participants, their data, or biological material

Policy information about studies with human participants or human data. See also policy information about sex, gender (identity/presentation), and sexual orientation and race, ethnicity and racism.

| | |
|---|---|
| Reporting on sex and gender | N/A |
| Reporting on race, ethnicity, or other socially relevant groupings | N/A |
| Population characteristics | N/A |
| Recruitment | N/A |
| Ethics oversight | N/A |

Note that full information on the approval of the study protocol must also be provided in the manuscript.

# Field-specific reporting

Please select the one below that is the best fit for your research. If you are not sure, read the appropriate sections before making your selection.

☒ Life sciences ☐ Behavioural & social sciences ☐ Ecological, evolutionary & environmental sciences

For a reference copy of the document with all sections, see nature.com/documents/nr-reporting-summary-flat.pdf

# Life sciences study design

All studies must disclose on these points even when the disclosure is negative.

| | |
|---|---|
| Sample size | The deep sequencing data were at least independently repeated twice. And all statistically analyzed experiments were independently repeated at least three times and determined according to standard molecular biology procedures. |
| Data exclusions | None |
| Replication | Biological replicates were used for the deep sequencing data. The ChIP-seq data in Extended Data Fig. 9d (without replicates) was validated by CUT&RUN data, and vice versa. At least three independent replicates were performed for statistically analyzed experiments. |
| Randomization | N/A |
| Blinding | N/A |

# Reporting for specific materials, systems and methods

We require information from authors about some types of materials, experimental systems and methods used in many studies. Here, indicate whether each material, system or method listed is relevant to your study. If you are not sure if a list item applies to your research, read the appropriate section before selecting a response.

## Materials & experimental systems

| n/a | Involved in the study |
| --- | --- |
| ☐ | ☒ Antibodies |
| ☐ | ☒ Eukaryotic cell lines |
| ☒ | ☐ Palaeontology and archaeology |
| ☒ | ☐ Animals and other organisms |
| ☒ | ☐ Clinical data |
| ☒ | ☐ Dual use research of concern |
| ☒ | ☐ Plants |

## Methods

| n/a | Involved in the study |
| --- | --- |
| ☐ | ☒ ChIP-seq |
| ☐ | ☒ Flow cytometry |
| ☒ | ☐ MRI-based neuroimaging |

# Antibodies

Antibodies used

Rabbit polyclonal antisera against hNDF; anti-hSpt16 (Cell Signaling, 12191S); anti-GAPDH (Cell Signaling, 5174S); anti-LEDGF (Proteintech, 25504-1-AP); anti-H3 (Cell Signaling, 4499); anti-H3K36me3 (Abcam, ab9050); anti-Rpb2/Pol II (Genetex, GTX102535); anti-Oct4 (Cell Signaling, 2750S); anti-GLYR1 (proteintech, 14833-A-AP); anti-Nestin (Cell Signaling, 33475S); ChIP-seq spike-in antibody (Active Motif, 61686); Donkey anti-Rabbit IgG (H+L) Highly Cross-Adsorbed Secondary Antibody, Alexa Fluor™ 555 (ThermoFisher, A31572); anti-rabbit IgG (H+L), F(ab')¬2 Fragment (Alexa Fluor 647 conjugate) (Cell Signaling, 4414S); anti-Vimentin (Cell Signaling, 5741); anti-RNA pol II CTD phospho Ser2 antibody (Abcam, ab237280); anti-RNA pol II CTD phospho Ser5 antibody (Active Motif, 61085).

Validation

Rabbit polyclonal antisera against hNDF: Tested in Western Blot and chromatin IP. Fei, J. et al. NDF, a nucleosome-destabilizing factor that facilitates transcription through nucleosomes. Genes Dev 32, 682-694 (2018). https://doi.org/10.1101/gad.313973.118
anti-hSpt16: Tested in Western Blot and chromatin IP. RRID: AB_2732025
anti-GAPDH: Tested in Western Blot. RRID: AB_10622025
anti-LEDGF: Tested in Western blot (Proteintech)
anti-H3: Tested in Western Blot. RRID: AB_10544537
anti-H3K36me3: Tested in Western Blot. RRID: AB_306966
anti-Rpb2/Pol II: Tested in Western Blot and chromatin IP. RRID: AB_1951313
anti-Oct4: Tested in immunofluorescence. RRID: AB_823583
anti-GLYR1: Tested in immunofluorescence. RRID: AB_10859775
anti-Nestin: Tested in Western Blot. RRID: AB_2799037
ChIP-seq spike-in antibody: Tested in chromatin IP. RRID: AB_2737370
Donkey anti-Rabbit IgG (H+L) Highly Cross-Adsorbed Secondary Antibody, Alexa Fluor™ 555: Tested in immunofluorescence. RRID: AB_162543
anti-rabbit IgG (H+L), F(ab')-2 Fragment (Alexa Fluor 647 conjugate): Tested in immunofluorescence. RRID: AB_10693544
anti-Vimentin: Tested in Western blot and Immunofluorescence. RRID: AB_10695459
anti-RNA pol II CTD phospho Ser2 antibody: Tested in Immunofluorescence (Abcam);
anti-RNA pol II CTD phospho Ser5 antibody: Tested in Immunofluorescence. RRID: AB_2687451.

# Eukaryotic cell lines

Policy information about cell lines and Sex and Gender in Research

Cell line source(s)

iPSC cell line GM25256, purchased from the Coriell Institute for Medical Research
Hela, HEK293T, and SW480 cells

Authentication

None of the cell lines were authenticated.

Mycoplasma contamination

Cell lines were not tested for mycoplasma contamination.

Commonly misidentified lines
(See ICLAC register)

No commonly misidentified cell lines were used.

# Plants

Seed stocks

N/A

Novel plant genotypes

N/A

Authentication

N/A

# ChIP-seq

## Data deposition

☒ Confirm that both raw and final processed data have been deposited in a public database such as GEO.

☒ Confirm that you have deposited or provided access to graph files (e.g. BED files) for the called peaks.

| | |
|---|---|
| Data access links<br>*May remain private before publication.* | https://www.ncbi.nlm.nih.gov/geo/query/acc.cgi?acc=GSE273678<br>Genome sequencing results are available on GEO under accession number GSE273678, GSE273679, GSE273680, and GSE273681 are publicly available. |
| Files in database submission | FASTQ and bigWig files for all ChIP-seq performed in this study |
| Genome browser session<br>(e.g. UCSC) | https://genome.ucsc.edu/s/ziweilirutgers/NDF%2DFACT%20Collection%2Dseperate%206 |

## Methodology

| | |
|---|---|
| Replicates | 2-5 |
| Sequencing depth | ≥ 20 million read pairs per sample<br>Number of Reads for each samples are listed below.<br>iPSC, NDF, ChIP_Rep1 19497569<br>iPSC, NDF, ChIP_Rep2 18296153<br>iPSC, Spt16, ChIP_Rep1 26023384<br>iPSC, Spt16, ChIP_Rep2 25132222<br>iPSC, Pol II, ChIP_Rep1 20305379<br>iPSC, Pol II, ChIP_Rep2 27163792<br>iPSC, Input 43154770<br>iPSC, NDF, ChIP_Con_Rep2 44721659<br>iPSC, NDF, ChIP_Hex_Rep1 49798214<br>iPSC, NDF, ChIP_Hex_Rep2 44050321<br>iPSC, Spt16, ChIP_Con_Rep1 39163867<br>iPSC, Spt16, ChIP_Con_Rep2 39613162<br>iPSC, Spt16, ChIP_Hex_Rep1 37919738<br>iPSC, Spt16, ChIP_Hex_Rep2 28861296<br>iPSC, Pol II, ChIP_Con_Rep1 37034310<br>iPSC, Pol II, ChIP_Con_Rep2 38533346<br>iPSC, Pol II, ChIP_Hex_Rep1 44004748<br>iPSC, Pol II, ChIP_Hex_Rep2 43605006<br>iPSC, LEDGF, ChIP_Con_Rep1 27134165<br>iPSC, LEDGF, ChIP_Con_Rep2 31075853<br>iPSC, LEDGF, ChIP_Hex_Rep1 32555943<br>iPSC, LEDGF, ChIP_Hex_Rep2 27590244<br>NDF-degron, Spt16, ChIP_DMSO1h_Rep1 27858945<br>NDF-degron, Spt16, ChIP_Aux1h_Rep1 27019702<br>NDF-degron, Spt16, ChIP_DMSO1h_Rep2 9253232<br>NDF-degron, Spt16, ChIP_Aux1h_Rep2 27697793<br>NDF-degron, Spt16, ChIP_DMSO1h_Rep3 31638338<br>NDF-degron, Spt16, ChIP_Aux1h_Rep3 41968610<br>NDF-degron, Spt16, ChIP_DMSO2h_Rep1 35993847<br>NDF-degron, Spt16, ChIP_Aux2h_Rep1 36676851<br>NDF-degron, Spt16, ChIP_DMSO2h_Rep2 50388156<br>NDF-degron, Spt16, ChIP_Aux2h_Rep2 42865323<br>NDF-degron, Spt16, ChIP_DMSO2h_Rep3 25126834<br>NDF-degron, Spt16, ChIP_Aux2h_Rep3 8336327<br>NDF-degron, Pol II, ChIP_DMSO2h_Rep1 88514789<br>NDF-degron, Pol II, ChIP_DMSO2h_Rep2 101037781<br>NDF-degron, Pol II, ChIP_Aux2h_Rep1 91731299<br>NDF-degron, Pol II, ChIP_Aux2h_Rep2 81334799<br>Spt16-degron, NDF, ChIP_DMSO4h_Rep1 28078730<br>Spt16-degron, NDF, ChIP_DMSO4h_Rep2 27575689<br>Spt16-degron, NDF, ChIP_Aux4h_Rep2 25262944<br>Spt16-degron, NDF, ChIP_Aux4h_Rep1 23137748<br>iPSC, NDF-WT, NDF, ChIP 29390351<br>iPSC, NDF-K, NDF, ChIP 27862531<br>iPSC, NDF-WT, Spt16, ChIP_Rep1 47814475<br>iPSC, NDF-WT, Spt16, ChIP_Rep2 43207389<br>iPSC, NDF-K, Spt16, ChIP_Rep1 47832626<br>iPSC, NDF-K, Spt16, ChIP_Rep2 43066968<br>NDF-degron, MNase_DMSO2h_Rep1 69562538<br>NDF-degron, MNase_Aux2h_Rep1 67487269<br>NDF-degron, MNase_DMSO2h_Rep2 85199559 |

NDF-degron, MNase_Aux2h_Rep2 79756405
NDF_WT, MNase 137013965
NDF_K161A, MNase 141917982
CPC, NDF-WT, RNA_seq_Rep1 15057749
CPC, NDF-WT, RNA_seq_Rep2 17048811
CPC, NDF-K, RNA_seq_Rep1 16370770
CPC, NDF-K, RNA_seq_Rep2 15596866
EpiC-FB, NDF-WT, RNA_seq_Rep1 16290706
EpiC-FB, NDF-WT, RNA_seq_Rep2 16517222
EpiC-FB, NDF-K, RNA_seq_Rep1 16007324
EpiC-FB, NDF-K, RNA_seq_Rep2 17535979
iPSC, NDF-WT, TT_seq_Rep1 41310192
iPSC, NDF-K, TT_seq_Rep1 47253876
iPSC, NDF-K, TT_seq_Rep2 47832290
iPSC, NDF-WT, DRB_TT_seq_0min_Rep1 42783378
iPSC, NDF-WT, DRB_TT_seq_10min_Rep1 45491724
iPSC, NDF-WT, DRB_TT_seq_10min_Rep2 36792511
iPSC, NDF-WT, DRB_TT_seq_20min_Rep1 41911359
iPSC, NDF-WT, DRB_TT_seq_20min_Rep2 35123866
iPSC, NDF-K, DRB_TT_seq_0min_Rep1 38242253
iPSC, NDF-K, DRB_TT_seq_0min_Rep2 41009986
iPSC, NDF-K, DRB_TT_seq_10min_Rep1 36425927
iPSC, NDF-K, DRB_TT_seq_10min_Rep2 33586462
iPSC, NDF-K, DRB_TT_seq_20min_Rep1 32218824
iPSC, NDF-K, DRB_TT_seq_20min_Rep2 30264401

Antibodies: See antibodies above

Peak calling parameters: no peak calling.

Data quality: Number of reads are listed above.

Software: See software above

# Flow Cytometry

## Plots

Confirm that:

☒ The axis labels state the marker and fluorochrome used (e.g. CD4-FITC).

☒ The axis scales are clearly visible. Include numbers along axes only for bottom left plot of group (a 'group' is an analysis of identical markers).

☒ All plots are contour plots with outliers or pseudocolor plots.

☒ A numerical value for number of cells or percentage (with statistics) is provided.

## Methodology

Sample preparation: For cell cycle analysis, the cells were dissociated with 1x TrypLETM-Select Enzyme (ThermoFisher, 12563029), about 2 million cells were washed with PBS and and resuspended in 300 µL of cold (4°C) PBS. Cells were fixed by the dropwise addition of 0.8 mL of cold (4°C) ethanol followed by incubation at 4°C for a minimum of 24 h. After fixation, the cells were pelleted and resuspended in 1 mL of PBS containing 50 µg/mL of propidium iodide (Sigma, P4170) and 10 µg/mL of RNase A (NEB, T3018). The cells were incubated for 30 min at 22°C and then subjected to flow cytometry analysis.
For cell apoptosis analysis, the dissociated cells were stained with Annexin V Conjugate (ThermoFisher, A23202) according to the manufacturer's instructions. The cells were then subjected to flow cytometry analysis.

Instrument: Cytek Aurora Spectral Cell analyzer

Software: The fcs files were processed with FlowJo

Cell population abundance: The filter retained about 40% of the original cells for cell cycle analysis (~10k out of 25k) and about 80% of the original cells for cell apoptosis analysis (~50k out of 60k).

Gating strategy: For cell cycle analysis, the debris were removed by filtering FSC-A and SSC-A values higher than 200000 and 600000, respectively. The singlets were further selected by filtering FSC-A and FSC-H with FSC-H value higher than 300000.
For cell apoptosis analysis, the debris were removed by filtering FSC-A and SSC-A values higher than 200000 and 200000, respectively. The singlets were further selected by filtering FSC-A and FSC-H with FSC-H value higher than 100000.

☐ Tick this box to confirm that a figure exemplifying the gating strategy is provided in the Supplementary Information.
