# Supplementary material for: Phase-separated NDF−FACT condensates facilitate transcription elongation on chromatin
Source: Nat Cell Biol. 2025 Sep 30;27(11):1938–51. doi: 10.1038/s41556-025-01778-8 (PMC12611769; doi:10.1038/s41556-025-01778-8)

Raw Figure 1b

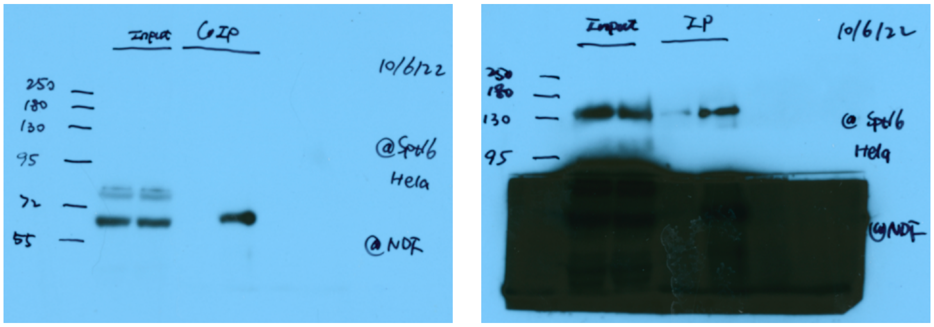

Raw Figure 1c

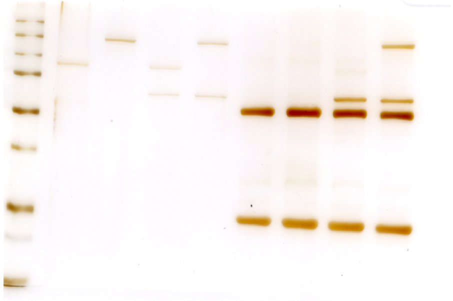

Raw Figure 1e

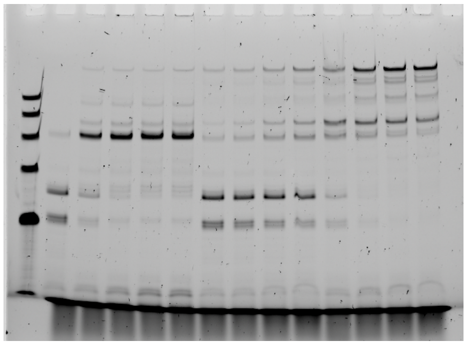

Raw Figure 2c

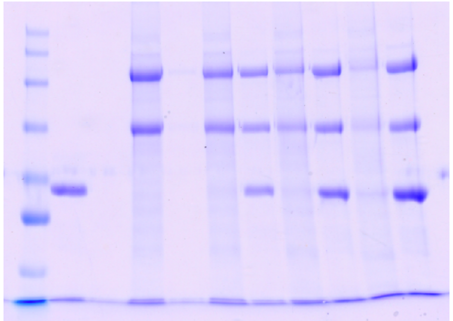

Raw Figure 2i

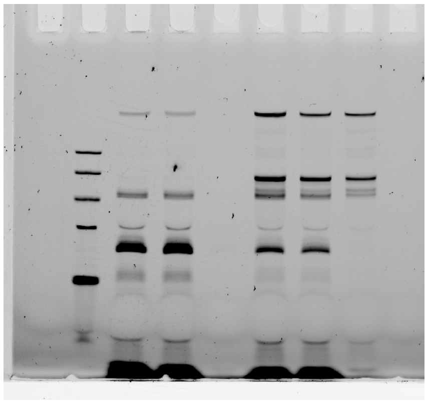

Raw Figure 2j

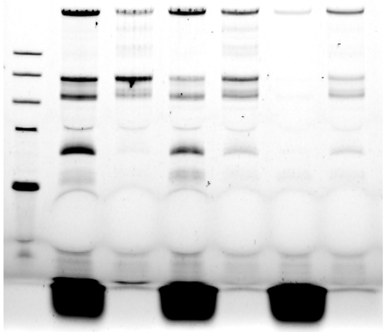

Raw Figure 4c

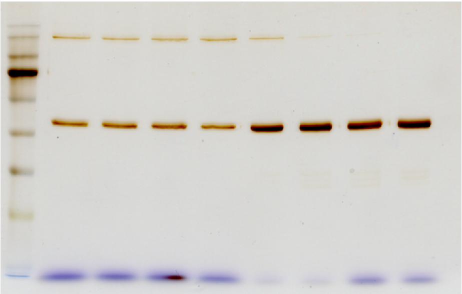

Raw Figure 4d

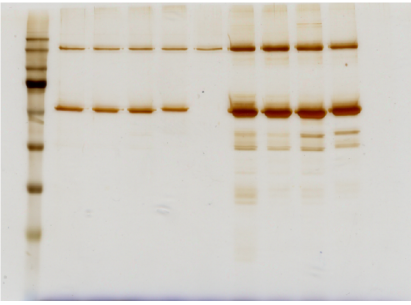

Raw Figure 4g

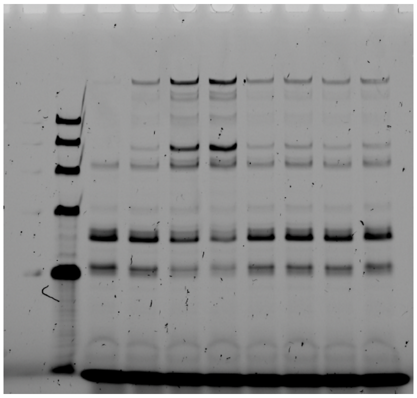

Raw Figure 4i

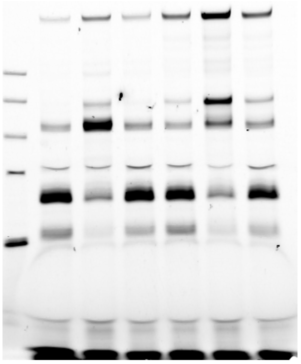

Raw Figure 6e

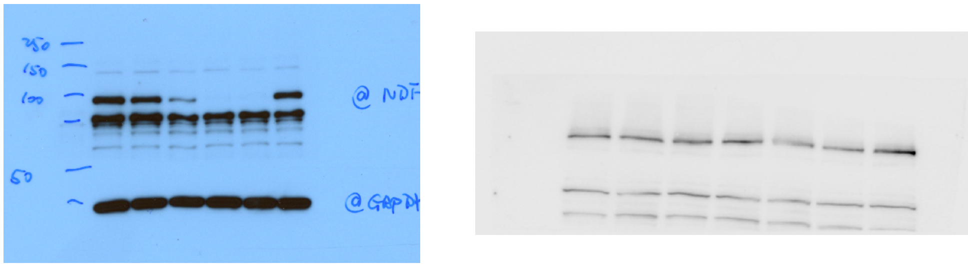

Raw Figure 7a

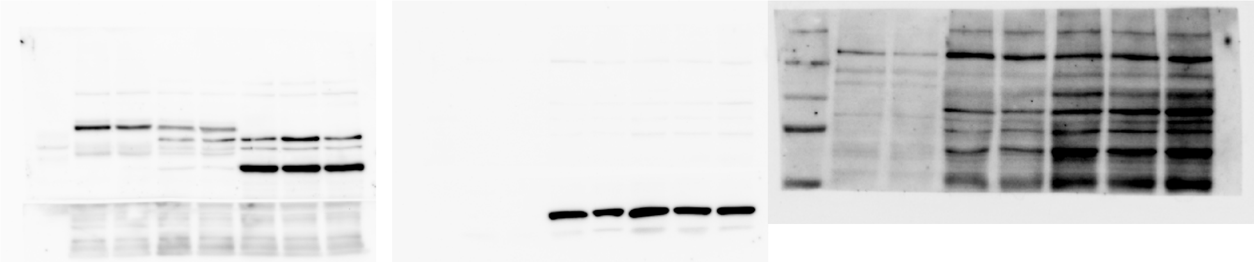

Raw Extended Data Fig. 1b

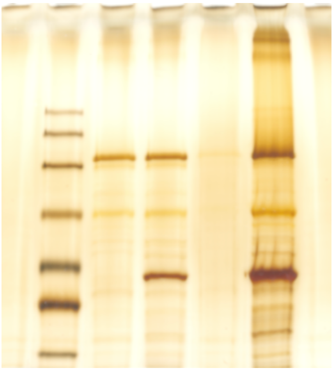

Raw Extended Data Fig. 4f

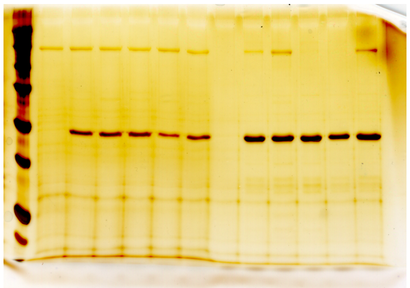

Raw Extended Data Fig. 1c

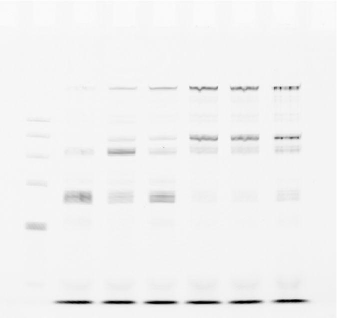

Raw Extended Data Fig. 4g

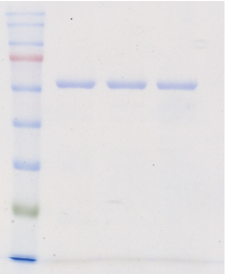

Raw Extended Data Fig. 4h

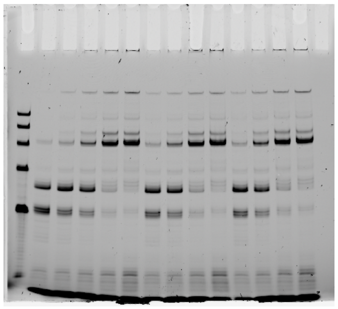

Raw Extended Data Fig. 2e

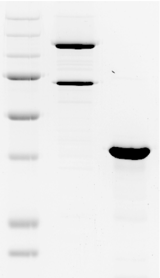

Raw Extended Data Fig. 4j

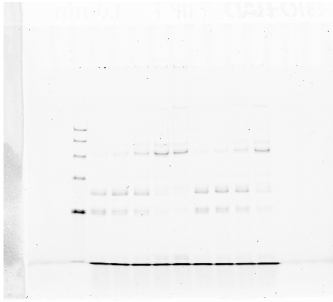

Raw Extended Data Fig. 4b

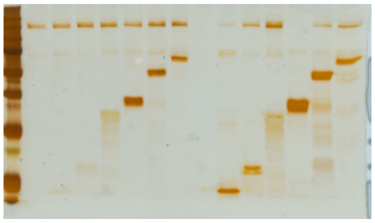

Raw Extended Data Fig. 4c

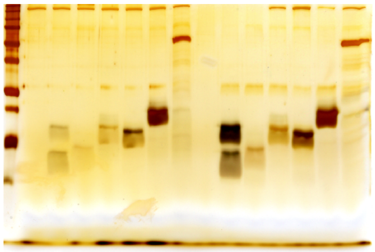

Raw Extended Data Fig. 5d

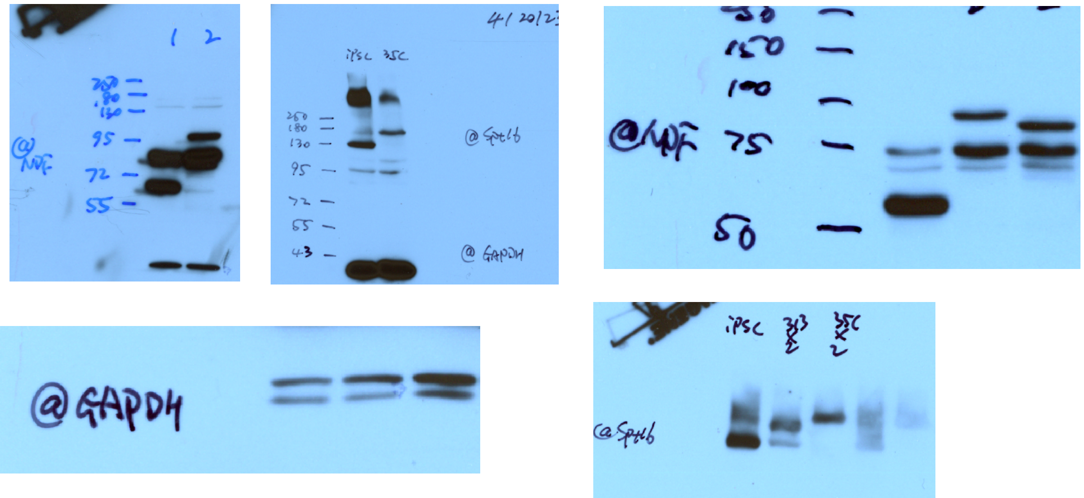

Raw Extended Data Fig. 5b-c

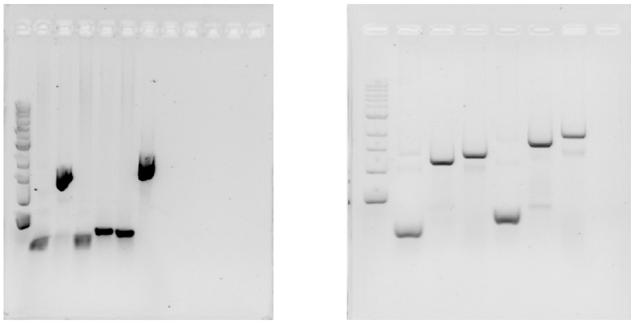

Raw Extended Data Fig. 5f

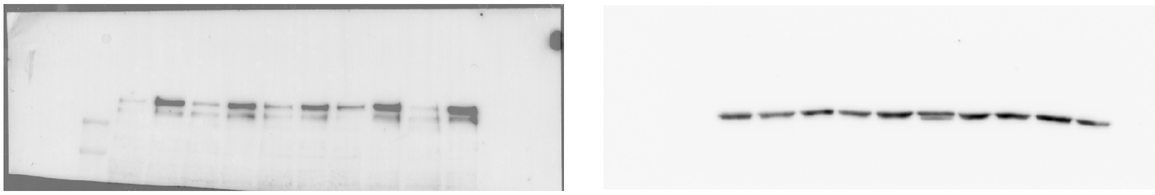

Raw Extended Data Fig. 6b

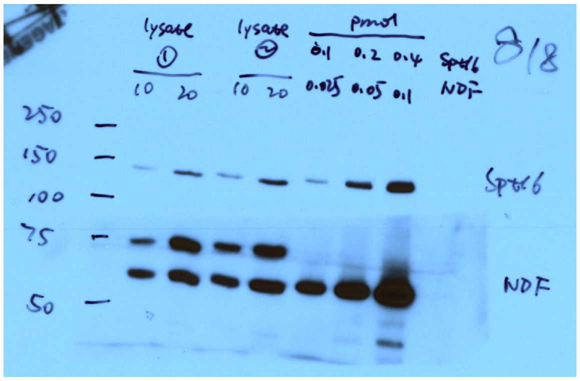

Raw Extended Data Fig. 6e

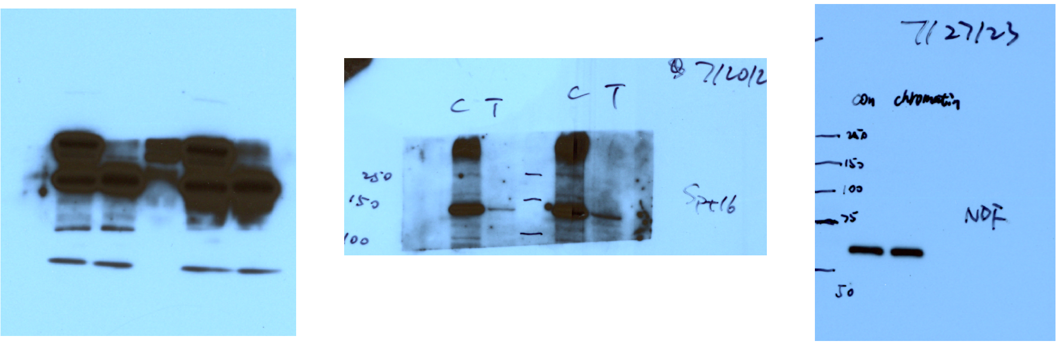

Raw Extended Data
Fig. 7g

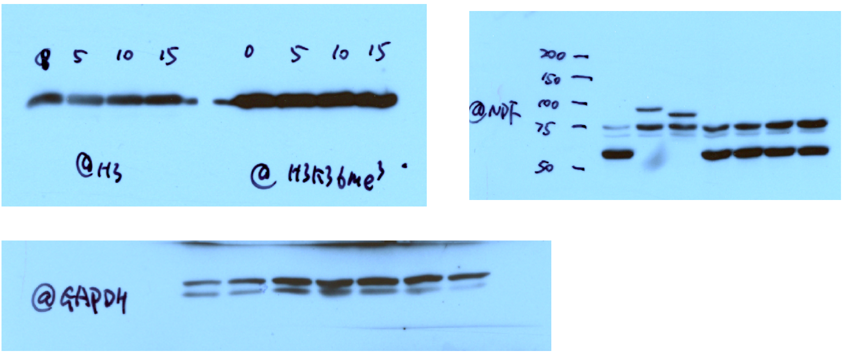

Raw Extended Data
Fig. 7h

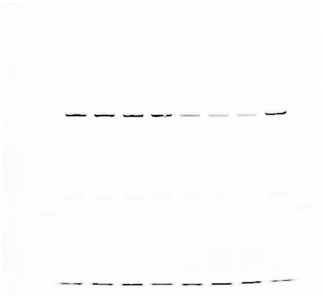

Raw Extended Data
Fig. 8a

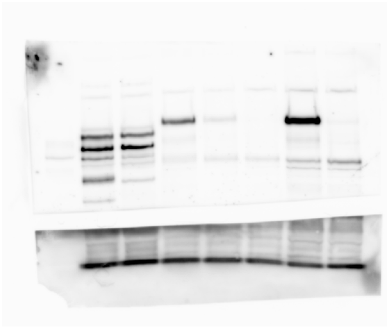

Raw Extended Data
Fig. 8e

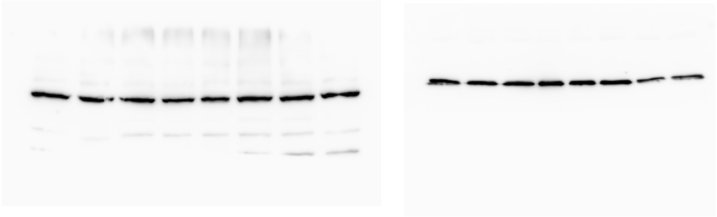

Supplement: Supplementary file 6 — Unprocessed gels and/or blots for all main and extended data figures. [file 41556_2025_1778_MOESM6_ESM.pdf]
